# Editorial: Augmented neuro-therapy with nanotechnology-based formulations: progress, opportunities and challenges

**DOI:** 10.3389/fphar.2024.1376878

**Published:** 2024-02-08

**Authors:** Mithun Singh Rajput, Viral Patel, Nilesh Prakash Nirmal, Dileep Kumar

**Affiliations:** ^1^ Department of Pharmacology, Institute of Pharmacy, Nirma University, Ahmedabad, Gujarat, India; ^2^ Department of Pharmaceutics, Ramanbhai Patel College of Pharmacy, Charotar University of Science and Technology (CHARUSAT), Anand, Gujarat, India; ^3^ Institute of Nutrition, Mahidol University, Salaya, Nakhon Pathom, Thailand; ^4^ UC Davis Comprehensive Cancer Center, Department of Entomology and Nematology, University of California Davis, Davis, CA, United States

**Keywords:** drug delivery, formulations, nanotechnology, neuropharmacology, neuroprotection

Substantial neurological disorders—neurodegenerative, neuropsychiatric, and neurotraumatic—have a severe damaging influence on quality of life and a significant health burden. Numerous acute and chronic neurological conditions are attributed to damage to neural cells resulting from oxidative stress, inflammation, and other molecular aetiologies (Degan et al., 2018). The strategies used in neurotherapeutics are difficult and complicated, and have the drawback of having synthetic chemicals’ side effects. A novel strategy is neuroprotection research that explicitly targets the use of safer nanomedicine. Numerous natural, semisynthetic, and synthetic substances have been shown to have neuroprotective properties. The therapeutic effects of these substances are mostly attributed to the molecular mechanisms that reduce oxidative stress, neuro-inflammation, and signal transduction associated with protein folding. These beneficial effects do, however, come with some side effects (Rajput et al., 2023). The most cutting-edge method for creating formulations is to mitigate or reduce specific toxicities. Nanotechnology-based formulations are the newest developments in this field. Key technological advantages of using nano-formulations as drug carriers include high stability, high carrier capacity, the ability to integrate both hydrophilic and hydrophobic substances, and the flexibility to deliver via various channels, such as oral route and other methods. Nano-formulations also offer the prospect of regulated or prolonged medication release from the matrix. Because of these properties, medications can be made more bioavailable, their dosage and frequency can be reduced, and any associated toxicity can be eliminated (Jeevanandam et al., 2016).

This Research Topic aimed to present new developments in innovative therapeutic approaches for the treatment of different neurological illnesses through the use of nano-formulations derived from natural, semi-synthetic, and synthetic chemicals. For their future work, neuroscientists, doctors, ethnopharmacologists, and researchers from academia and business will have access to more comprehensive knowledge.

Augmented neuro-therapy with nanotechnology-based formulations Research Topic includes four papers covering different aspects of neuroprotection with an amalgamation of natural products with nanotechnology. Two of these articles report on the isolation and applications of different bioactive compounds from herbs. Furthermore, these herbs have been shown to exert neuroprotective benefits. Motivated by literature, using microwave aided extraction Callizot et al. created a green one-step process that yields an extract of *Huperzia serrata* (Thunb.) Trevis known as NSP01. In traditional Asian medicine, *H. serrata* is frequently used to treat a variety of nervous system ailments, such as dementia, schizophrenia, and cognitive impairment. The original neuropharmacological activity and chemical profile of the conventional extract are preserved in the microwave-assisted green extract. The precise mix of three main components—huperzine A and two phenolic acids, caffeic acid and ferulic acid—is what gives NSP01 its neuroprotective effect. It has been demonstrated that ferulic acid and caffeic acid enhance the neuroprotective effects of huperzine A. Crucially, ferulic acid and caffeic acid alone do not enhance huperzine A’s acetylcholinesterase inhibitory activity, which is what causes its unfavourable side effects. All of these experimental results showed that NSP01 is a very promising plant extract that can be used to prevent memory loss and Alzheimer’s disease.

Due to its positive benefits on brain function and cognition, the herb *Centella asiatica* is employed in traditional Chinese and Ayurvedic medicine. In a study, Speers et al. employ metabolomic analysis of cortical tissue from 5xFAD mice given varying amounts of water extract of *Centella asiatica* (WCA) in an effort to clarify the processes behind the effects of WCA in the mouse brain. WCA significantly changed metabolic pathways linked to purine metabolism, nicotinate and nicotinamide metabolism, and glycerophospholipid metabolism in at least three of the four treatment groups (5xFAD or wild-type, male or female). These results provide new light on other plausible mechanisms for the WCA-induced enhancement of cognition, such as brain-derived neurotrophic factor modulation and nicotinamide adenine dinucleotide upregulation. The aetiology of Alzheimer’s disease has been linked to these metabolic pathways, underscoring the therapeutic promise of WCA in this neurodegenerative condition.

The therapeutic drugs currently available can delay the symptoms of Huntington’s disease (HD), but consistent usage can have long-term adverse effects. The low occurrence of side effects in herbal drugs like engeletin has garnered interest. By inhibiting the Keap1/NRF2 pathway, engeletin has been demonstrated to lessen mitochondrial dysfunction and regulate inflammation. Its poor permeability and solubility, however, prevent it from getting to the intended location. A theoretical formulation of engeletin-nanostructured lipid nanocarriers was put up by Smriti et al. to treat Huntington’s disease (HD) with better distribution across the blood-brain barrier and higher bioavailability. Due to its composition resembling the natural lipids found in the body, lipid solubility, and increased bioavailability, this formulation may enable free passage over the blood-brain barrier and aid in the treatment or prevention of HD. Here, a review by Bose et al. primarily focuses on the many methods used for successful nanotheranostics applications of complex chemicals related with cyclodextrin (CD) in the field of therapeutics. Through the use of CDs as nanoparticles, a highly effective method of generating nanocomplexes and a drug delivery system, there may be possible nanotheranostics treatments for a variety of cancers, including those of the brain, lung, colon, cervix, and many more. Through ongoing research and cutting-edge techniques, nanotheranostics presents a promising route for revolutionising different cancer treatments, including brain cancer treatment.

Nanomedicines are avant-garde above traditional remedies for central nervous system (CNS) ailments and have been linked to matters with treating neurological disorders. The development of nanoformulations of several neuroprotective medications, including curcumin, edaravone, nerve growth factors, etc. has been linked to nanotechnology; nevertheless, little information is now available regarding their side effects (Naqvi et al., 2020). With the potential to completely change how we approach CNS-targeted therapeutics, the use of nanotechnology in CNS drug delivery has a bright future ahead of it. This is because it can be used to nanoengineer drugs or carriers to target specific cells, diffuse within brain tissue, cross the blood-brain barrier, or activate signalling systems to deliver neuro therapeutics.
